# Research progress on multivalent genetically engineered vaccines against enterotoxigenic *Escherichia coli*

**DOI:** 10.3389/fimmu.2026.1736447

**Published:** 2026-05-18

**Authors:** Hongli Li, Zongfa Yang, Yuhan She, Chongli Xu, Wenqiao Ding

**Affiliations:** 1 College of Medical Technology,Chongqing Medical and Pharmaceutical College, Chongqing, China; 2 College of Biology and Food Engineering, Jilin University of Chemical Technology, Jilin, China

**Keywords:** diarrhea, enterotoxigenic *Escherichia coli*, enterotoxins, fimbriae, vaccine

## Abstract

This article systematically elaborates on the etiological characteristics, epidemiology and current status of prevention and control of diarrhea caused by enterotoxigenic *Escherichia coli* (ETEC). ETEC is an important pathogenic bacterium responsible for diarrhea in young animals and its pathogenicity is closely associated with specific adhesins and enterotoxins. The article focuses on the serotype diversity of ETEC and its relationship with pathogenicity. It also analyzes the differences in susceptibility among various animal hosts and age groups. Furthermore, it outlines the epidemiological features of its transmission through contaminated water and food. In response to the drug resistance issues resulting from antibiotic overuse, the article highlights vaccination as the most effective current prevention and control strategy. It also summarizes the research progress and application potential of multivalent vaccines, genetically engineered vaccines and novel live-vector vaccines targeting fimbrial antigens and enterotoxins. This review aims to offer important theoretical insights for a deeper understanding of the pathogenic mechanisms of ETEC and the development of highly effective and broad-spectrum vaccines.

## Introduction

Enterotoxigenic *Escherichia coli* (ETEC) is an important pathogen that causes diarrhea in animals, including newborn piglets, calves, lambs and weaned piglets (1–4). Animals infected with ETEC often die from severe watery diarrhea and rapid dehydration, with high morbidity and mortality rates (5, 6).People are generally susceptible to ETEC infection. Patients and carriers are the primary sources of infection and they can contaminate the surrounding environment and spread rapidly. Most infected individuals develop severe diarrhea due to food and water contaminated with ETEC. As a member of the Enterobacteriaceae genus, ETEC is a Gram-negative bacterium and does not form spores. Some strains possess a polysaccharide capsule. The optimum growth conditions for ETEC were at 37°C and a pH range of 7.2 to7.4 (7). ETEC adheres to the surface of the small intestine without damaging or invading the intestinal mucosal epithelial cells. It disrupts electrolyte secretion and causes diarrhea primarily by producing enterotoxins (8). It is one of the primary pathogens responsible for “traveler’s diarrhea” in developed countries. ETEC is a common cause of “adult cholera syndrome” and an important pathogen responsible for pediatric diarrhea (9). Its incidence rate is second only to that of rotavirus. Worldwide, ETEC can cause diarrhea in hundreds of millions of people each year, particularly infants and children in developing countries (10).

## The relationship between ETEC pathogenicity and serotype

ETEC can cause intestinal or parenteral infections in many animals, particularly diarrhea in young animals. Young animals infected with ETEC often die from rapid dehydration due to severe watery diarrhea, which results in high morbidity and mortality rates (11). However, there are differences between different species and different ages. There are approximately 40 typical ETEC serotypes and ETEC infectious diarrhea is associated with the pathogen’s characteristics, environmental conditions and host susceptibility (12). Only infection with ETEC, which carries the adhesion factor and produces a certain dose of the toxin, can lead to diarrhea. If the farrowing environment is unclean, particularly with ETEC contaminating the sow’s teats and skin, piglets are at high risk of infection, primarily through nursing (13). Although antibodies in colostrum and milk have a certain protective effect on piglets, ETEC infection can be caused by low levels of specific antibodies and insufficient suckling of individual piglets. ETEC diarrhea primarily occurs in intensive pig farms and breeding facilities. ETEC frequently causes diarrhea in newborn piglets aged 0 to 6 days, as well as in weaned piglets aged 3 to 6 weeks. There is no obvious seasonality and although it is more common during the low-temperature, wet and cold seasons. After over 20 years of extensive research, the relationship between diarrhea and ETEC serotypes in young livestock has been clearly elucidated. There are approximately40 typical ETEC serotypes that are prevalent worldwide which include 12 O antigen groups. Currently, the primary O antigen groups identified are 8, 9, 20, 45, 60, 64, 101, 115, 139, 141, 148 and 157. Certainly, in a specific geographical region and over a certain period of time, the dominant strains of ETEC that can be isolated from different countriesare different (14).

### ETEC virulence factor

ETEC has a relatively complex antigen composition, which mainly includes O-antigen, K antigen, H antigen and fimbrial antigen. The O antigen is a complex of polysaccharide, phospholipid and protein, which is present in the cell wall of bacteria. It primarily acts as a toxic component in bacteria and is a heat-resistant bacterial antigen. Its antigenicity cannot be destroyed by heating at 100 °C or 121 °C and it exhibits resistance to ethanol and dilute acid (15). Thus, the O antigen plays a crucial role in the classification of *E. coli* serotypes. The specificity of O antigen is located on the polysaccharide side chain of LPS. There are two kinds of O antigen-specific polysaccharide side chains: neutral polysaccharide chain and acidic polysaccharide chain. There were eight kinds of neutral sugars, including glucose, galactose, mannose and rhamnose. There are seven kinds of acidic sugars, mainly including glucuronic acid and galacturonic acid. The epitope of the O antigen is determined by the type, location, arrangement and spatial conformation of the monosaccharides on the specific polysaccharide chain, which serves as the material basis for the specificity of the O antigen. The K antigen, a polysaccharide or protein antigen on the bacterial surface, influences the virulence of enterotoxigenic *E. coli* and is primarily found in fimbriae, envelopes or capsules. The H antigen is a heat-resistant flagellar protein antigen that plays an important role in bacterial motility. It primarily exists in flagella and possesses good antigenic properties. Ethanol can gradually destroy its antigenicity.

ETEC produces virulence factors such as adhesin, enterotoxin, endotoxin and hemolysin. Enterotoxin and adhesin play important roles in both the pathogenesis and immunology of the infection.With the help of these adhesion proteins, ETEC adheres to the epithelial cells of the intestinal mucosa of the host, thereby multiplying and producing a large number of enterotoxins, which cause pathological changes in the intestinal mucosal epithelial cells and lead to diarrhea in young animals. The protein fimbriae of ETEC are fibrous and are coated on the surface, which can protect the bacteria from complement-mediated killing and phagocytosis by macrophages. The main adhesin antigens are K88(F4), K99 (F5), 987p (F6), F41, F42 and so on. K88 is the most common adhesin antigen, which mainly includes K88ac, K88ab, K88ad and K88ae (16). In addition, F18 fimbriae have emerged as another significant adhesin, particularly associated with post-weaning diarrhea (PWD) in piglets. Notably, epidemiological shifts in fimbrial prevalence have been observed in recent years. Since around 2017, detection rates of F18 have shown a steady increase, while the prevalence of K88 (F4) has declined in certain regions, a trend reported in both China and the United States (17, 18). This highlights the evolving landscape of ETEC adhesins and underscores the importance of including F18 in the development of broadly protective multivalent vaccines.

All known adhesins of ETEC are proteins that are heat-resistant. Their biological and immunological activities can be destroyed by heating at 100°C. However, when heated at 60°C for 20 to 30 minutes, they can be detached from the cells without being inactivated.The specific fimbriae of ETEC are different from the type I fimbriae commonly found in *E. coli*. Except for the 987P antigen, all the others can agglutinate red blood cells.The hemagglutination activity of K88, K99 and F41 was not inhibited by D-mannose whereas the agglutination reaction of type I fimbriaeon erythrocytes was inhibited by D-mannose. Adhesin antigens are composed of hundreds of identical protein subunits. In SDS-PAGE, the purified K88, K99, 987P and F41 antigens all exhibited protein bands. However, their subunit molecular weights differed. The amino acid sequence analysis of K88, K99, 987P and F41 revealed that the amino acid composition of the three K88 serovariants was significantly different. All variants contained various common amino acids with the exception of cysteine. There are 15 differences in amino acid composition between K88ab and K88ac, most of which were related to charged amino acid residues. Consequently, the C-terminus of the K88 peptide is quite hydrophobic, whereas the middle section is highly hydrophilic and contains most of the basic amino acids (19). K88 has almost no homology with K99 or F41, but there is limited homology between F41 and K99. All types of adhesins exhibit good antigenicity and can stimulate the body to produce highly effective antibodies.

### Enterotoxin

Enterotoxin is a type of exotoxin that can be categorized into heat-stable enterotoxin (ST) and heat-labile enterotoxin (LT) based on its mechanism of action and thermal stability (20). These two enterotoxins can be produced separately or concurrently and the production of both toxins is controlled by plasmids. According to antigenicity and host differences, ST is divided into ST_1_ and ST_2_. ST_1_ includes ST_1a_ and ST_1b_. ST_1_ has been identified as a small peptide consisting of 18 or19 amino acids. When heated at 100°Cfor 30 minutes, it remains active but has no immunogenicity (21). LT is composed of an A subunit of 28 kDa combined with five B subunits of 11.5 kDa. Both the LT and the B subunits exhibit good immunogenicity (22). ST_1_ contains 13 highly conserved amino acid sequences that retain the full toxicity of ST_1_ and possess receptor protein binding ability similar to that of natural toxins. In the conserved region, Cys5-Cys10, Cys6-Cys14 and Cys9-Cys17 form three intramolecular disulfide bonds. Of these, the disulfide bond composed of Cys6-Cys14 is crucial for ST_1_ toxicity, while the other two disulfide bonds maintain the complete spatial structure of ST_1_. The ST_1_ molecule is acidic and contains a high proportion of hydrophobic amino acids. At lowionic strength, it readily polymerizes with alkaline hydrophobic polypeptides. ST_1_ is resistant to heat, acids, alkalis, a variety of organic solvents and surfactants and it cannot be hydrolyzed by various proteases such as amylase, trypsin, lipase, Streptomyces protease and glycosylase. However, ST_1_ is highly sensitive to oxidants and reducing agents, which can destroy disulfide bonds, resulting in the loss of biological activity of ST after treatment with these agents. ST_1_ by itself is not immunogenic. However, when conjugated with macromolecules, it can exhibit immunogenicity.The toxicity of ST_1_ is closely related to the presence of three disulfide bonds within the molecule. If any disulfide bond is destroyed, its toxicity will be reduced by at least 250 times. If two intramolecular disulfide bonds are destroyed, the toxicity of ST_1_ may be completely lost. When TGT encoding the 14th cysteine (Cys) in ST_1_ gene is replaced by AGT, the toxic effect of ST_1_ is significantly weakened. This indicates that the cysteine at position 14 is indispensable for the activity of ST_1_ (23). LT isoelectric point is 6.9 and has antigenicity. It can be inactivated by acid, pepsin, trypsin, amylase and pancreatic lipas. Additionally, LT can be precipitated using ammonium sulfate. The research results indicated that 20-30% of the enterotoxigenic *E. coli* strains that caused colibacillosis in piglets were LT^+^/ST^-^ and 30-40% of the ETEC isolates were LT^+^/ST^+^ and the LT^-^/ST^+^ strains accounted for nearly 50% (24). Compared with LT, ST plays a more important role in young livestock diarrheal diseases caused by enterotoxigenic *E.coli*, which poses a great threat to domestic animal health. Therefore, it is of great significance to study the various properties, pathogenic mechanism and immunogenicity of ST at the molecular level for controlling diarrheal diseases caused by ST.

### Pathogenesis of ETEC’s virulence factors

The pathogenicity of ETEC is fundamentally governed by the specific interaction between its adhesins and host intestinal receptors, which determines bacterial tropism in terms of both host species and developmental stage. In swine, ETEC strains utilize a repertoire of adhesins including K88 (F4), K99 (F5), 987P (F6), F41 and F18 for colonization. Among these, F18 fimbriae are a major etiological agent of post-weaning diarrhea (PWD) (25). The expression of corresponding host receptors is developmentally regulated: receptors for K88 (F4) are present from birth, enabling infection in both neonatal and weaned piglets, whereas full expression of the F18 receptor typically occurs around 20 days of age, explaining its predominant association with PWD (26). For ruminants such as calves and lambs, prevalent ETEC adhesins include K99, F41 and F17a (27).

The binding specificity of adhesins dictates their host range. Strains expressing K99 or F41 can adhere to the small intestine of pigs, cattle and sheep due to the widespread presence of their receptors across these species. In contrast, adhesins like K88, 987P and F18 show a more restricted tropism, primarily colonizing the porcine small intestine (28). Furthermore, within the porcine intestine, different adhesins exhibit distinct tissue localization: K88-positive bacteria primarily colonize the duodenum, K99 or F41 strains are found in the jejunum and ileum and 987P bacteria reside in the ileum.

Upon entering a susceptible host, ETEC utilizes its fimbriae to bind to specific receptors on the microvilli and brush border of small intestinal epithelial cells. This adhesion, involving approximately 30 to 40 bacteria per cell, enables the bacteria to resist intestinal peristalsis and fluid flow, facilitating colonization. Subsequent bacterial multiplication and the release of enterotoxins lead to the pathological changes that cause diarrhea. The proteinaceous nature of adhesins confers strong immunogenicity. Immunization with whole cells containing adhesins or with purified adhesin antigens can elicit high-potency antibody responses, forming the theoretical basis for adhesin-based vaccine development.

**Figure 1:**
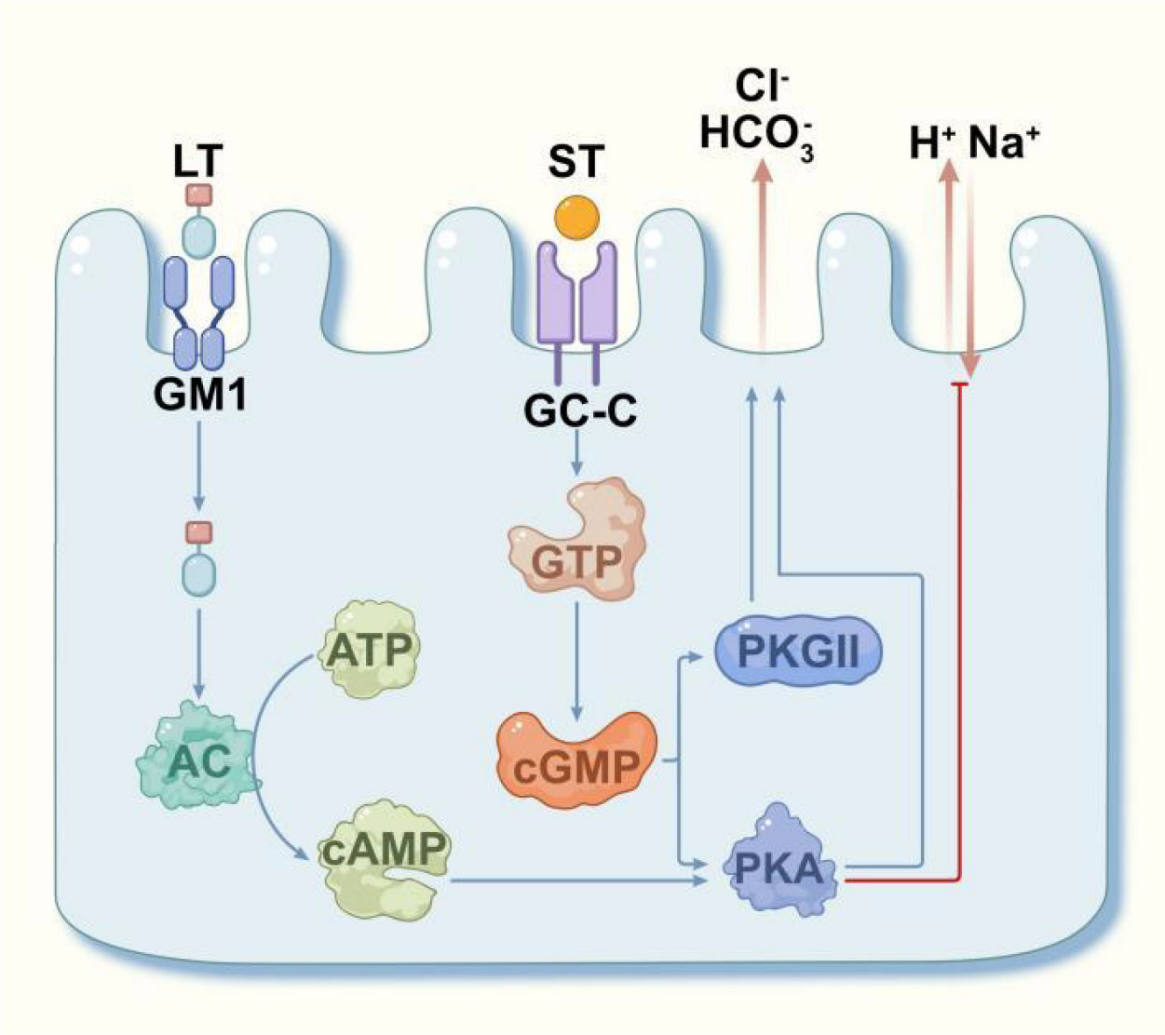
Mechanism of action of ETEC toxins on intestinal epithelial cells.

Enterotoxin is a direct pathogenic factor that causes diarrhea. It has been confirmed that ST_1_ can activate guanylate cyclase (GC) in small intestinal epithelial cells, resulting in an increased intracellular cGMP concentration (29). Subsequently, cGMP directly activates protein kinase G II (PKGII) and protein kinase A(PKA), thereby disrupting electrolyte secretion and leading to diarrhea. Disulfide compounds inhibit this enzyme, thereby preventing ST_1_ from pathogenesis. The bioactivity of ST_1_ is tissue-specific and can increase cGMP in ileal epithelium by 10 times while in the lower colon by only 1.8 times. It does not exhibit activity in other tissues and organs. This specificity may be associated with receptor distribution. Mg^2+^ and Mn^2+^ plasma can enhance the effect of ST_1_. LT or its B subunit exhibits good immunogenicity. The A subunit is the active component of the toxin. The B subunit binds to GM1 ganglioside on intestinal mucosal cells, facilitating pore formation. The released A subunit enters the cell through pores to activate adenylate cyclase, thereby increasing the concentration of cAMP in the host cell and activating cAMP-dependent protein kinases (30). This triggers excessive secretion of water and electrolytes into the intestinal cavity by the intestinal mucosal cells, which increases the fluid volume in the intestinal cavity and leads to diarrhea (**Figure 1**).

### Immunoprophylaxis using ETEC polyvalent vaccines

Immunoprevention is a more promising and sustainable strategy than drug therapy for diarrhea in young animals caused by ETEC. Early research on genetically engineered vaccines laid the foundation for the development of polyvalent vaccines, while studies over the past decade have made significant breakthroughs in antigen design, delivery systems and breadth of protection.

Early research successfully constructed various bivalent vaccines targeting specific adhesins or enterotoxins using genetic engineering techniques, providing important insights and technical validation for subsequent development. For example, the K88-K99 dual-antigen engineered bacterial vaccine, created by recombining plasmids containing K88 and K99 genes, induced high levels of colostral antibodies in sows, offering passive protection to suckling piglets. Similarly, bivalent vaccines such as K88ac-LT_B_ and K99-F41 were subsequently developed and demonstrated certain protective efficacy in animal models. For enterotoxins, key early attempts to address the weak immunogenicity of heat-stable enterotoxin (ST) involved genetic fusion or chemical conjugation of ST with the B subunit of heat-labile enterotoxin (LT). These pioneering studies confirmed the feasibility of combining multiple critical virulence factor antigens through genetic engineering. However, these early vaccines generally had a narrow protective spectrum, and the challenges of effective detoxification and presentation of ST remained incompletely resolved.

### *E. coli* K88/K99 bivalent genetically engineered vaccine

The researchers cut pTK90 and pTK56 plasmids containing the *K88* and *K99* genes with the restriction endonuclease *Bam*HI enzyme and then linked them with T_4_ DNA ligase. Subsequently, they were transformed into the *E. coli* C600 strain and the genetically engineered strain carrying the plasmid pTK8899 was screened (31). The K88-K99 bivalent genetically engineered vaccine was administered to pregnant sows approximately 21 days prior to delivery and the levels of antibodies to K88 and K99 in the colostrum were assessed after sows gave birth. The results indicated the presence of highly reactive antibodies to K88 and K99 in the colostrum. Piglets could effectively resist ETEC infection after sucking colostrum. The K88-K99 bivalent genetically engineered vaccine prepared by high-density fermentation and antigenic protein vaccine process did not require formaldehyde treatment. The antigen protein retained its natural spatial structure, which was conducive to inducing antibody production. At the same time, this freeze-dried preparation could be stored for an extended period, which was very beneficial for storage and transportation. Using genetic engineering technology, the researchers developed K88-K99 bivalent genetic engineering vaccine. This vaccine was capable of producing both K88 and K99 surface antigens simultaneously and its expression level had reached or exceeded that of wild donor strains. When pregnant sows were immunized with this vaccine, a good immune response was induced. Piglet obtained two protective antibodies K88 and K99 by sucking colostrum, which produced local immune protection in intestinal mucosa. Thus prevented the adhesion of K88, K99 fimbrial *E. coli*, thereby preventing diarrhea.

### K88ac-LT_B_ bivalent genetically engineered vaccine

Plasmid pMM031 containing *K88ac* gene and plasmid pPMC4 containing *LT_B_* gene were respectively digested by restriction enzyme *Bam*HI to construct recombinant plasmid pMM085. The recombinant strain MM-3 was created by transforming this plasmid into the C600 strain. The results indicated that the antigen production of K88ac and LT_B_ in MM-3 was essentially equivalent to that of the parent strains (32). The toxicity test revealed that MM-3 could be utilized as a live vaccine to prevent piglet diarrhea. Considering that resistance genes commonly used in genetic engineering might cause biological hazards, they were not suitable for long-term use. For this purpose, the *LacZ* gene was inserted into a plasmid vector containing the LT_B_ subunit gene. A recombinant plasmid containing a tetracycline resistance gene was constructed to express both the LT_B_ subunit and β-galactosidase (33). Subsequently, the *K88ac* gene was inserted into the tetracycline resistance gene of the recombinant plasmid, thus inactivating the tetracycline resistance gene. The test results showed that the recombinant plasmid MM-6 could express LT_B_ and K88ac antigens. Moreover, LacZ gene replaced tetracycline resistance gene as a good screening marker. Compared with MM-3, MM-6 also exhibited a good immune protection effect. Recombinant plasmids containing the *LT_B_* and *K88ac* antigen genes were transformed into attenuated strains of Salmonella choleraesuis using the bacterial plasmid transformation technique. The transformed bacteria retained the morphological, biochemical and antigenic characteristics of Salmonella. Simultaneously, the LT_B_ and K88ac antigens were stably and efficiently expressed, exhibiting characteristics of both Salmonella cholerae suis and enterotoxigenic *E. coli*. The results provided an effective candidate strain for the development of a bivalent genetically engineered vaccine against these two bacteria. Pregnant sows were immunized via oral and intramural injection, respectively. The results indicated that a high antibody response against the antigens was formed in the serum and whey of the immunized sows.

### K99-F41 bivalent genetically engineered vaccine

The plasmid pMGK99 containing *K99* gene was digested by *Bam*HI enzyme and linked to *F41* gene to construct recombinant plasmid pMG611 (34). The transgenic strain MM-7 was transformed into *E. coli* HB101. The molecular weight of the K99 and F41 antigens was the same as that of the wild strains. An ELISA assay revealed that the expression level of the K99 and F41 fimbrial antigens in MM-7 was higher than that in the wild strains. Active immunity can be induced by subcutaneous immunization of pregnant ewes with MM-7. Lambs acquire passive immunity by ingesting the colostrum of the ewes. The immunoassay results indicated that MM-7 exhibited a strong protective effect. There were significant differences in mortality and the number of diarrhea days between the experimental group and the control group, suggesting that MM-7 possessed good immunogenicity.

### ST_1_-LT_B_ bivalent genetically engineered vaccine

Fusion studies of ST and LT were first conducted at the protein level. Some basic rules regarding the fusion of ST and LT were obtained. ST and LT were initially coupled with carbonized diimide at a ratio of 100:1. The antigenicity of the conjugate product was greater than 80% of the original toxin and the toxicity of ST and LT was reduced to as low as 0.15% of the original toxin. The study demonstrated a good protective effect against ST and LT in immunized mice. Subsequently, 39% of ST and 61% of LT were chemically coupled to immunize mice and the toxicity of ST was reduced to 0.06% of the original toxin (35). The antibody titers of serum IgG and mucosal IgA against the enterotoxin increased 4–7 times after immunizing the mice, indicating that the fusion of ST and LT possessed certain immunogenicity. Subsequent researchers conjugated the synthetic STa to a carrier protein and immunized animals using Foley’s adjuvant to elicit an antibody response (36). Antibody peaks can persist for several weeks. Newborn animals exhibited a protective effect against ETEC attacks. Therefore, using LT as the carrier protein for ST renders ST a complete antigen, capable of inducing an immune response to both ST and LT. The experiment demonstrated that it was non-toxic. The LT enteroendocrine IgA titer increased in correlation with the immune dose and provided the same level of immune protection as ST. These results suggested that partial LT_B_ had a lesser impact on the immunogenicity of ST in fusion protein molecules compared to full-length LT_B_. Therefore, the fusion protein exhibits enhanced ST immunogenicity, which is of great importance for further understanding the interactions between the various components of the fusion. After gene fusion, proteins can be continuously obtained through bacterial culture, which is superior to chemical crosslinking. However, the fusion of ST and LT is still being explored. Through various methods of ST and LT gene fusion studies, we should continue to improve the fusion methods and pathways so that the ETEC vaccine can play a key role in the prevention and control of piglet diarrhea more efficiently.

The LT_B_ subunit has good immunogenicity and studies on chemical cross-linking with ST had shown that LT_B_ could act as a carrier protein for ST and endowed it with immunogenicity. Consequently, the *LT_B_-ST* gene fusion is being pursued as a research direction for polyvalent vaccine candidate strains. Studies on the fusion of LT_B_ and ST genes had indicated that the fusion at both the N-terminal and C-terminal ends did not significantly impact the effect. The ST was placed at the 5’ end and LT_B_ at the 3’ end for gene fusion and molecular hybridization confirmed that the expressed product could bind to anti-LT_B_ antibodies. LT_B_ was placed upstream of the ST gene to facilitate gene fusion and the expression product tested positive by ELISA following the use of appropriate splicers. Animal experiments demonstrated that the fusion protein was non-toxic and possessed the immunogenicity of both ST and LT_B_. Previous studies had indicated that a fusion protein created by linking pro-ST, the precursor of ST, to LT_B_ was also immunogenic. This fusion protein induced an immune response and exhibited reduced toxicity associated with ST (37).

In the study of gene fusion, the junction between ST and LT_B_ influenced the immune activity of various parts of the fusion protein. For example, when a 9bp junction was used, the expression product was negative in the ELISA test. Using 21bp (encoding Asp-Pro-Arg-Val-Pro-Ser-Ser oligonucleotides) and 24bp (encoding His-Asp-Pro-Arg-Val-Pro-Ser-Ser oligonucleotides) as linkers, all parts of the fusion protein were found to be immunogenic. Therefore, it was very important to have a suitable joint for all parts of the fusion protein to maintain good activity. In addition, the secretory expression of the fusion protein was also very important for constructing ETEC vaccine candidate strains. However, so far, the fusion protein of ST and LT_B_ had not achieved satisfactory secretory expression. Existing studies could only observe the secretion of expression products into the periplasm, but not into the extracellular environment. The researchers found that the 18.5kDa protein product was converted from a precursor molecule of 20.8kDa, which was clustered within the cell. Subsequent studies confirmed that the wild-type ST was secreted into the extracellular environment through two processes. The pre-pro-ST consisting of 72 amino acid residues was processed by the signal peptidase to remove 19 amino acid residues. The pro-ST was secreted into periplasm and then processed into mature ST with 18–19 amino acids. Furthermore, studies on *LT_B_-ST* gene fusion had revealed that ST could also gain immunogenicity due to the carrier protein function of the cholera toxin B subunit (CT-B) (38). The CT-B signal peptide was crucial for the secretion of the fusion protein. This undoubtedly offered a valuable reference for research on the secretion and expression of LT_B_ and ST fusion proteins. In previous studies on gene fusion between LT_B_ and ST, the activities of various components of the expression products were impacted to varying extents. In particular, the decrease of ST activity was more obvious. Our research team successfully fused the *ST* gene with the *LacZ* gene (which encodes β-galactosidase) through repeated tandem fusion and discovered that the expression product from the tandem *ST* gene exhibited enhanced antigenicity and non-toxicity, providing an effective approach for future studies on gene fusion between LT_B_ and ST (39).

Currently, the vaccines primarily used in the market included K88-K99, K88ac-LT_B_ and K88-K99-LT_B_ genetically engineered vaccines. Due to the unresolved toxicity of ST, the direct cathartic factor responsible for piglet colibacillosis, the immune effect is not optimal. Therefore, it is necessary to develop a new and effective genetically engineered vaccine to ultimately prevent piglet colibacillosis. *E. coli*-induced colibacillosis is primarily treated with drugs and prevented using various whole-cell and specific fimbrial vaccines. However, due to the complex and diverse serotypes of the pathogenic bacteria and the limited broad-spectrum protection of traditional vaccines, the effectiveness of both drug treatment and vaccine prevention is often unsatisfactory. A large number of studies had shown that the colonization factors of *E. coli* causing colibacillosis in piglets vary among different serotypes. However, most of these *E. coli* produce ST_1_ and LT toxins. In addition, considering that K88ac and K99 are also dominant pili, researchers constructed the K88ac-LT_B_ bivalent genetically engineered vaccine, which achieved a good immune effect. Nevertheless, they did not solve the worldwide puzzle of ST_1_ immunogenicity (40).

Our team constructed the *Pro-ST_1_-LacZ* fusion gene and studied the fusion protein it expressed. The results indicated that the fusion protein had lost the toxic properties of ST_1_. The antibody produced by the immunized mice was capable of neutralizing the natural toxicity of ST_1_, suggesting that the LacZ protein had imparted immunogenicity to ST_1_. At the same time, we successfully constructed the Pro-ST_1_-LT_B_ fusion gene and studied its expression and immunogenicity. The results indicated that the recombinant strain could express the ST_1_-LT_B_ fusion protein, which accounted for 33.21% of the total protein content. Whole cultures of the inactivated strain BL21 (DE3) (pXETSLT1) were utilized as bivalent genetically engineered vaccine against colibacillosis in newborn piglets caused by enterotoxigenic *E. coli* enterotoxin. The animal experiments demonstrated that effective immune protection was achieved in preventing colibacillosis in piglets (41).

Subsequently, we amplified the *K88ac* gene, the *ST_1_* mutant gene and the *LT_B_* gene from the *E. coli* C83902 plasmid using polymerase chain reaction and gene site-specific mutation. Next, the recombinant expression plasmid pXKST3LT5, containing the *K88ac-ST_1_-LT_B_* fusion gene, was constructed using DNA recombination technology (42). Finally, the recombinant expression plasmid was transformed into the recipient bacterium BL21 (DE3) and the recombinant strain BL21 (DE3) (pXKST3LT3) was obtained. The restriction digestion identification and DNA sequence analysis confirmed that the recombinant expression plasmid pXKST3LT5 contained the *K88ac-ST_1_-LT_B_* fusion gene and the gene sequence and reading frame were both correct. The SDS-PAGE and ELISA results indicated that the recombinant strain could express the K88ac-ST_1_-LT_B_ fusion protein. Gel thin-layer scanning analysis revealed that the fusion protein accounted for 33.53% of the total protein content. Animal experiments demonstrated that the K88ac-ST_1_-LT_B_ fusion protein expressed by the engineered strain was non-toxic. The immunogenicity test confirmed that the fusion protein possessed good immunogenicity and the induced serum was capable of neutralizing the toxicity of natural ST_1_. This suggested that the engineered strain BL21(DE3)(pXKST3LT5) could potentially be used as a candidate strain for preventing yellow diarrhea in piglets. At the same time, because fimbrial antigen K99 is also one of the major virulence factors, we amplified the *K88ac* gene, the *ST_1_* mutant gene and the *LT_B_* gene from the plasmid of *E. coli* C83902 and the *K99* gene from *E.coli* C83539 using PCR and gene site-specific mutation technology. Recombinant strain BL21 (DE3) (pXKKSL4) containing *K88ac-K99-ST_1_-LT_B_* fusion gene expression vector was constructed by DNA recombination technique (43). The recombinant plasmid pXKKSL4 contained the *K88ac-K99-ST_1_-LT_B_* fusion gene by enzyme digestion and DNA sequence analysis and the gene sequence and reading frame were correct. The culture conditions of the recombinant strain were optimized and the efficient expression was realized. The ELISA results indicated that the K88ac-K99-ST_1_-LT_B_ fusion protein expressed by the recombinant strain was recognized by the ST_1_ monoclonal antibody, LT_B_, K88ac and K99 antibodies.The safety test of the recombinant strain demonstrated that the fusion protein had lost the toxicity of the natural ST_1_ enterotoxin. Animal experiments showed that the immune protection rate was more than 90%. The results indicated that the recombinant strain could be used as a candidate strain for genetically engineered vaccine against diarrhea of newborn piglets. This can prevent piglet colibacillosis caused by ETEC through its enterotoxin and fimbriae. It has significant academic and application value and is expected to yield substantial economic and social benefits.

### Novel polyvalent and live-vector vaccine strategies

To overcome the limitations of early vaccines, such as their narrow protective spectrum and suboptimal immunogenicity, recent research has focused on designing more complex multivalent fusion antigens and exploring novel live-vector delivery systems, aiming to induce more comprehensive and efficient mucosal and systemic immune responses.

A core strategy in modern vaccine design is the construction of single multimeric fusion proteins intended to simultaneously elicit immune responses against multiple adhesins and enterotoxins. Studies have fused FaeG (the major subunit of K88), FedF (the adhesin of F18 fimbriae) with the detoxified LT A_2_ subunit and the pentameric LT_B_ to create a 1FaeG-FedF-LT_A2_:5LT_B_ complex. This ingenious design integrates the functions of toxin neutralization (anti-LT) and adhesion blockade (anti-K88 and anti-F18). Animal experiments demonstrated that it induces broad-spectrum neutralizing antibodies and provides effective protection against K88ac/LT challenge. This offers a design blueprint for vaccines targeting multiple ETEC fimbrial types prevalent in pig farms (44).

Given that clinical isolates often produce multiple enterotoxins simultaneously, vaccines targeting all major enterotoxins (LT, STa, STb) are particularly important. Through protein engineering, researchers introduced the serine 63 mutation (LTR192G) into native LT toxin to eliminate toxicity and then fused it with detoxified mutant forms of STa (STa_A13Q_) or STb, constructing LT_192_-STa_13_ and LT_192_-STb monomeric immunogens. Oral administration of attenuated *E. coli* simultaneously expressing these two immunogens in mice induced specific mucosal IgA and systemic IgG antibodies against LT, STa and STb, with the antibodies exhibiting *in vitro* neutralizing activity (45).

More recent studies have further optimized this type of fusion. Researchers constructed a recombinant protein containing LT_A1_-STa_13_-STb-LT_A2_-LT_B_-STa_13_-STb, aimed at enhancing immunogenicity and stability. This design inserts STa_13_-STb fragments between the LT_A1_/_A2_ subunits and at the C-terminus of LT_B_, forming a bicistronic structure. It is stably expressed in attenuated *E. coli* and can induce specific serum IgG and fecal sIgA against LT, STa and STb in mice, while also eliciting a Th2-biased cellular immune response (46).

Beyond designing single fusion proteins, the use of genetically engineered attenuated bacteria as vectors to co-express multiple heterologous antigens represents another efficient polyvalent vaccine strategy. Researchers used attenuated Shigella flexneri 2a as a backbone. By deleting its O-antigen synthesis gene (*rfbF*) and invasion-associated genes (*ipaBC*), the strain was rendered non-pathogenic and lost its serotype specificity. Subsequently, a gene cassette expressing a detoxified ETEC enterotoxin fusion protein (LT_B_-ST_N12S_) was stably inserted into its invasion plasmid, creating a candidate vaccine strain named ShigETEC.This vaccine can induce cross-protection against multiple Shigella serotypes in mouse models and generate antibodies capable of neutralizing both LT and ST toxins, demonstrating its potential for broad-spectrum antibacterial applications (47).

### Novel live-vector and delivery system

In addition to optimizing the antigens themselves, the use of safe live bacterial carriers for mucosal delivery is an ideal strategy for stimulating local intestinal immunity. Utilizing food-grade *Lactococcus lactis* as a live-vector is a highly attractive novel vaccine platform. A recent study fused mutated LT _A_ subunit (dmLT_A_), LT_B_ subunit and the key K88 fimbrial protein FaeG to surface display peptides, constructing three recombinant lactic acid bacteria. Administered orally as a mixture, this vaccine not only effectively prevented F4^+^ ETEC infection in weaned piglets devoid of maternal antibodies but also, when given to sows in late pregnancy, conferred protection to suckling piglets via colostral antibodies. This strategy highlights the unique advantages of live-vector oral vaccines in stimulating mucosal immunity, avoiding injection stress and providing full-cycle protection through a “sow-piglet” immunization program (48).

Besides LAB, other carefully attenuated bacteria are also used as vaccine vectors. For instance, attenuated *E. coli* strains constructed by deleting virulence genes can be safely used for oral immunization and effectively express and deliver ETEC antigens. Studies had detoxified enterotoxins through point mutations (LTR192G) and constructed attenuated strains harboring combinations of multiple detoxified enterotoxin genes (LT, ST_a_, ST_b_), thereby developing safe and effective oral vaccine candidates that simultaneously target multiple toxins (45). Other researchers utilized gene repair and λ-Red homologous recombination systems to insert polyvalent enterotoxin fusion genes into the pseudogene *yaiT* site of an attenuated *E. coli* O142 strain. The constructed oral vaccine strain *ER-T* demonstrated good safety, stability and immunogenicity in a mouse model. It effectively induced mucosal and systemic immune responses and provided protection against toxin challenge (46). These studies confirm the significant value of attenuated live bacterial vectors in mimicking natural infection processes and efficiently activating mucosal immunity.

## Conclusions

Enterotoxigenic *E. coli* is an important pathogen that causes diarrhea in young animals, including piglets, calves and lambs. It is also one of the significant pathogens responsible for the high incidence and mortality rates of bacterial diarrhea in animals. Following infection with ETEC, individuals often succumb to severe watery diarrhea and rapid dehydration, which can be fatal. The high morbidity and mortality rates associated with ETEC seriously impact human health and the sustainable development of the livestock industry. ETEC has become a prominent cause of infant diarrhea in developing countries, and it is also a factor in diarrhea cases among children, adults and tourists. Patients and carriers serve as the primary sources of infection, contaminating the surrounding environment and facilitating rapid spread. Numerous outbreaks of diarrhea have been linked to water, food, milk and beverages contaminated with ETEC.

ETEC is a member of the family Enterobacteriaceae and is a Gram-negative bacterium. ETEC possesses a complex antigenic composition, which includes the cell O antigen, the flagellar H antigen and the capsule K antigen. It exhibits various serotypes across different geographical regions. ETEC produces several toxins, such as adhesins, enterotoxins, endotoxins, haemolysins and so on. Enterotoxins and adhesins play important roles in both the pathogenesis and immunology of disease. The primary adhesin antigens include K88, K99,987p, F41 and F42. ETEC fimbriae bind to various receptors on the intestinal mucosal epithelium, allowing the bacteria to adhere to the intestinal epithelial cells and avoid rapid excretion due to the constant peristalsis of the animal’s digestive tract. This adhesion creates conditions conducive to the massive growth and reproduction of the bacteria and the production of enterotoxins, which can cause disease. Adhesins are protein-based antigens with strong immunogenic properties. Immunizing animals with cells containing adhesins or with purified adhesin antigens can efficiently produce the corresponding antibodies.With the aid of these adhesion proteins, ETEC colonizes the epithelial cells of the host’s intestinal mucosa and produces a significant amount of enterotoxin. This enterotoxin induces pathological changes in the intestinal mucosal epithelial cells, ultimately resulting in diarrhea in piglets. Enterotoxins can generally be divided into two categories: heat-stable enterotoxins (ST) and heat-labile enterotoxins (LT). Strains have the capability to produce ST or LT alone, or both. Enterotoxin plays an important role in ETEC diarrhea, so the study of its pathogenesis and immunogenicity is of great significance for the prevention and control of diarrheal diseases caused by *E. coli*. The results indicated that 20% - 30% of the diarrhea cases were LT^+^/ST^-^; 30% - 40% were LT^+^/ST^+^; and LT^-^/ST^+^ accounted for approximately 50%. Therefore, compared to LT, ST appears to play a more significant role in young livestock diarrheal diseases caused by enterotoxigenic *E. coli*. ST is further categorized into ST_1_ and ST_2_. ST_1_ includes ST_1a_ and ST_1b_. The genes encoding the ST enterotoxin are located on the plasmid and ST is not immunogenic. LT consists of one A subunit with a molecular weight of 28 kDa, combined with five B subunits, each with a molecular weight of 11.5 kDa. Both the complete LT molecule and its B subunits exhibit good immunogenicity. The treatment for ETEC diseases primarily involves the use of drugs and vaccines. These include toxoid vaccinesor LT_B_ subunit vaccines, which mainly target anti-enterotoxins. Additionally, monovalent or polyvalent inactivated whole vaccines and subunit vaccines are primarily designed to induce antiadhesion immunity. Avoiding the economic losses caused by this disease has become an urgent issue that needs to be addressed. In recent years, the overuse of antibiotics has led to the presence of drug residues and the development of bacterial resistance. Consequently, vaccination is considered the most effective method for controlling the disease.Currently, both commercial and experimental vaccines designed to prevent ETEC infections are contributing not only to the advancement of existing inactivated whole vaccines, subunit vaccinesand genetically engineered vaccines but also to the development of emerging DNA vaccines, transgenic vaccines and other novel vaccine technologies. However, the majority of vaccines currently used in the market are traditional inactivated vaccines, which come with their own set of limitations. Although genetically engineered vaccines such as K88-K99 and K88ac-LT_B_ are available, they have not addressed the issues of immunogenicity and toxicity associated with the primary pathogenic factor, ST_1_ enterotoxin. Consequently, these vaccines are unable to stimulate the body to produce an effective preventive response. In response to this challenge, our research group utilized gene site-specific mutation technology to amplify the mutant *ST_1_* gene, as well as the *K88ac*, *K99* and *LT_B_* genes. Then, we constructed the recombinant strain BL21(DE3)(pXKKSL4). The expression vector pXKKSL4 contained a K88ac-K99-ST_1_-LT_B_ fusion gene. Additionally, the fusion protein was highly expressed in the recipient bacteria, with the expression level comprising 35.72% of the total protein content in the bacteria. ELISA tests confirmed that the fusion protein was capable of binding to ST_1_ monoclonal antibody, as well as to LT_B_, K88ac and K99 antibodies. Intragastric test in rats showed that K88ac-K99-ST_1_-LT_B_ fusion protein had lost the activity of ST_1_ enterotoxin. The immune protection test indicated that both the strain vaccine and the inclusion body crude extract vaccine, when used with aluminum hydroxide as an adjuvant, exhibited a good immune effect. The immune protection rate of 1 MLD challenge dose strain was 98% and that of 1 MLD challenge dose inclusion body crude extract was 96%. The immune protection rate of the control group of K88-LT_B_ bivalent vaccine was 88%, indicating that the constructed K88ac-K99-ST_1_-LT_B_ vaccine had a good immune effect. The aforementioned studies demonstrated that our research group successfully developed a candidate strain for a tetravalent genetically engineered inactivated vaccine against major virulence factors of ST_1_, LT_B_, K88ac and K99. This vaccine not only addresses the toxicity issue associated with ST_1_ enterotoxin but also imparts immunogenicity. Additionally, increasing the expression of K99 pili can further augment the immune response. Consequently, this vaccine, which incorporates both enterotoxins and fimbriae, can be utilized to prevent diarrhea caused by ETEC. It has the potential to effectively control the incidence of diarrhea and establish a strong foundation for the future development of the ETEC genetically engineered inactivated vaccine, potentially yielding significant economic and social benefits.
